# Supplementary figures and images for: A NaV1.8FlpO mouse enabling selective intersectional targeting of low threshold C fiber mechanoreceptors and nociceptors
Source: Front Mol Neurosci. 2025 Jun 3;18:1574219. doi: 10.3389/fnmol.2025.1574219 (PMC12172507; doi:10.3389/fnmol.2025.1574219)

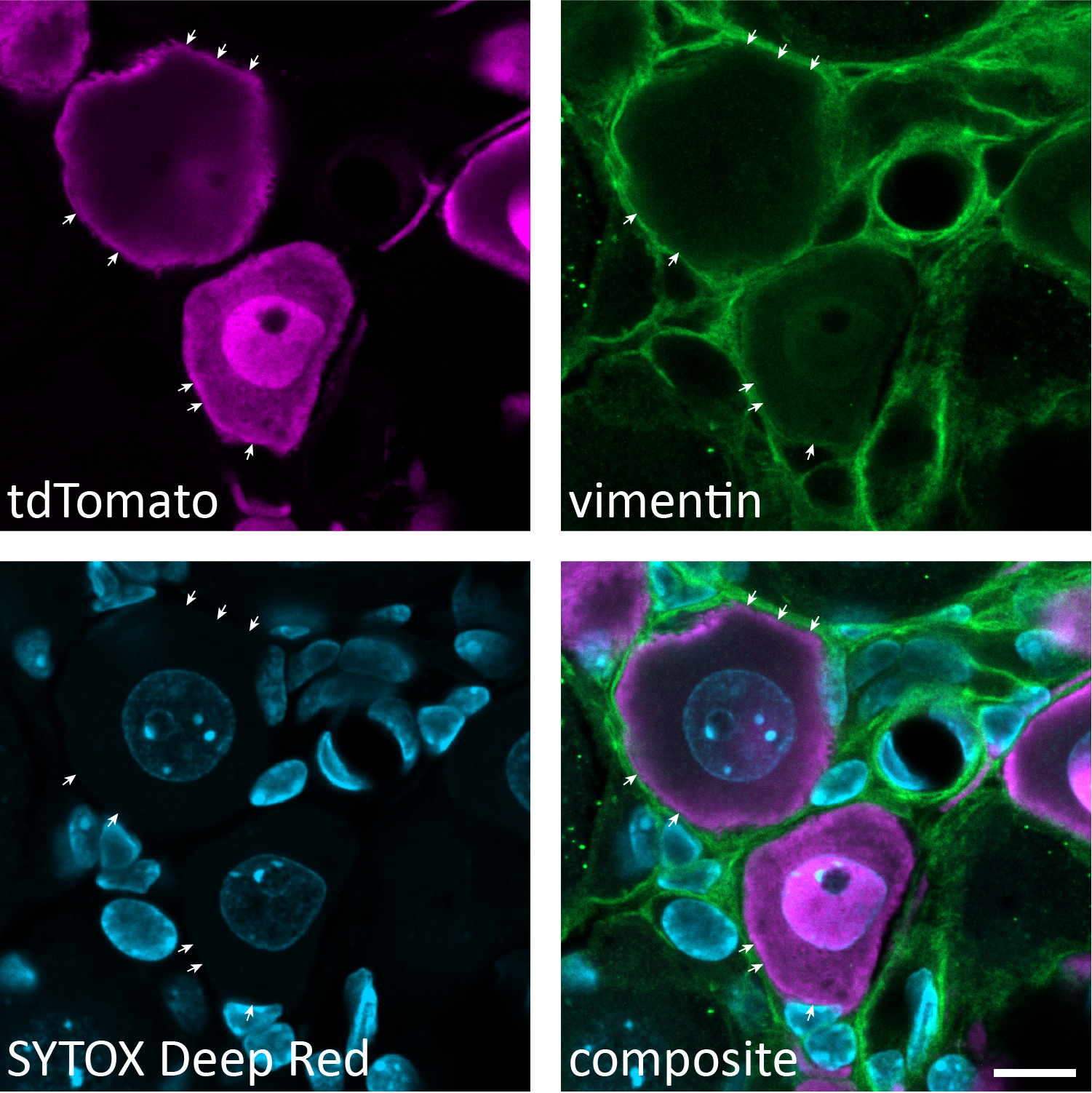

Supplement: SUPPLEMENTARY FIGURE S1 — tdTomato is not localized to satellite glial cells in DRGs of NaV1.8FlpO; Ai65F mice. Shown is an example view of tdTomato+ neurons, labeled using rabbit anti-RFP, exhibiting enrichment of labeling at the edge of the cells (indicated by arrows). Co-labeling with vimentin as a satellite glial cell marker shows that this enrichment of tdTomato labeling is restricted to neurons and does not localize to adjacent satellite glial cell. SYTOX Deep Red was used as nuclear staining. Scale bar, 10 μm. Images are from a maximum intensity projection of 5 optical sections at 0.260 μm separation acquired using a 40x/1.3 objective and 24 μm pinhole. [file Image_1.jpeg]

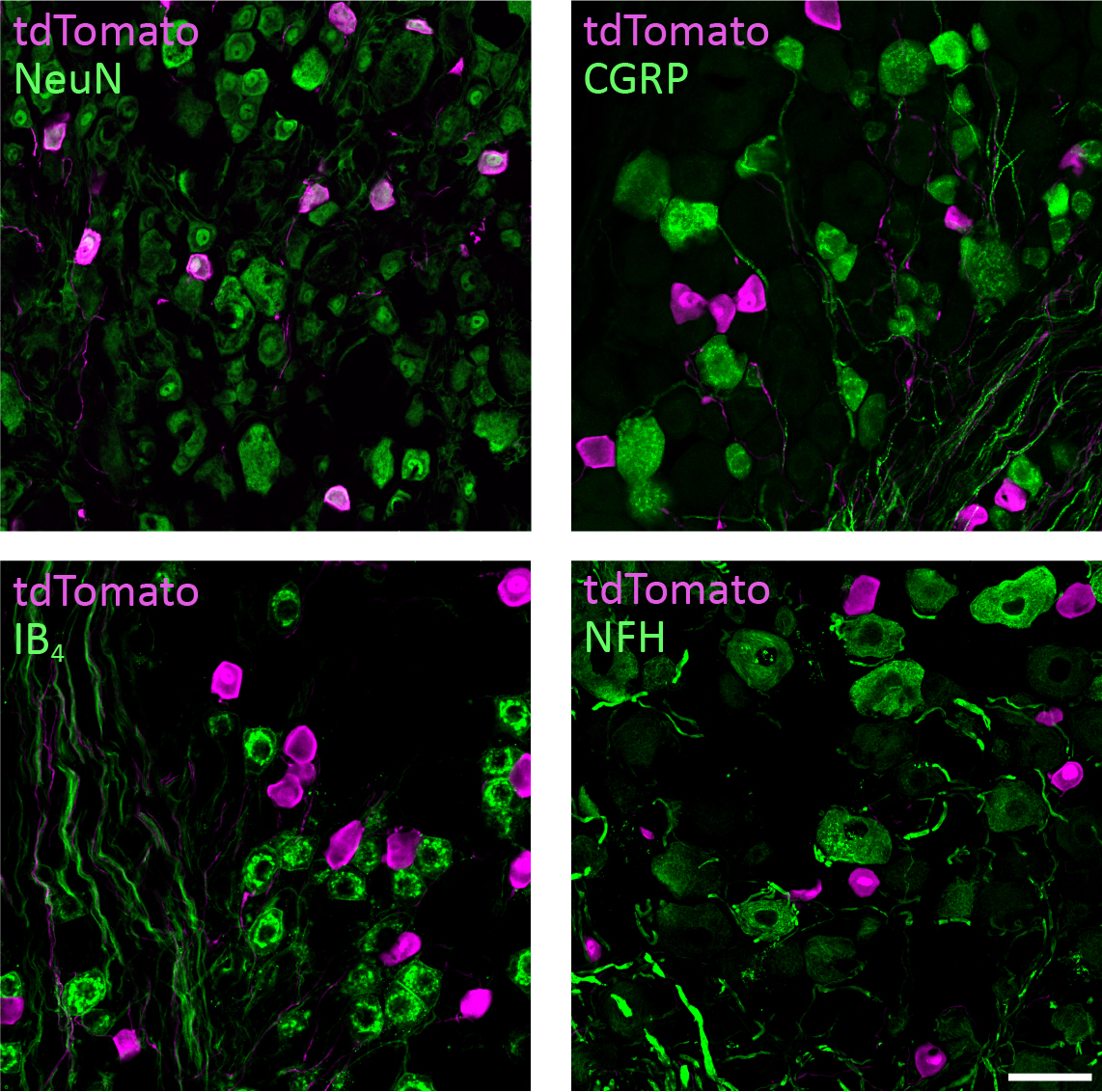

Supplement: SUPPLEMENTARY FIGURE S2 — Co-localization of tdTomato and cell markers in DRGs from ThCreERT2; NaV1.8FlpO; Ai65 mice. Shown are example L3-5 DRG sections labeled for NeuN, CGRP, IB4 binding and NFH. Note lack of co-localization of tdTomato with the nociceptive markers CGRP and IB4 binding, as well as with the A fiber marker NFH. Scale bar is 50 μm, valid for all panels. Images are single optical sections obtained using a 20x/0.8 objective with pinholes of 24 μm (top left panel), 27 μm (top right and bottom left panels), or 32 μm (bottom right panel). [file Image_2.jpg]

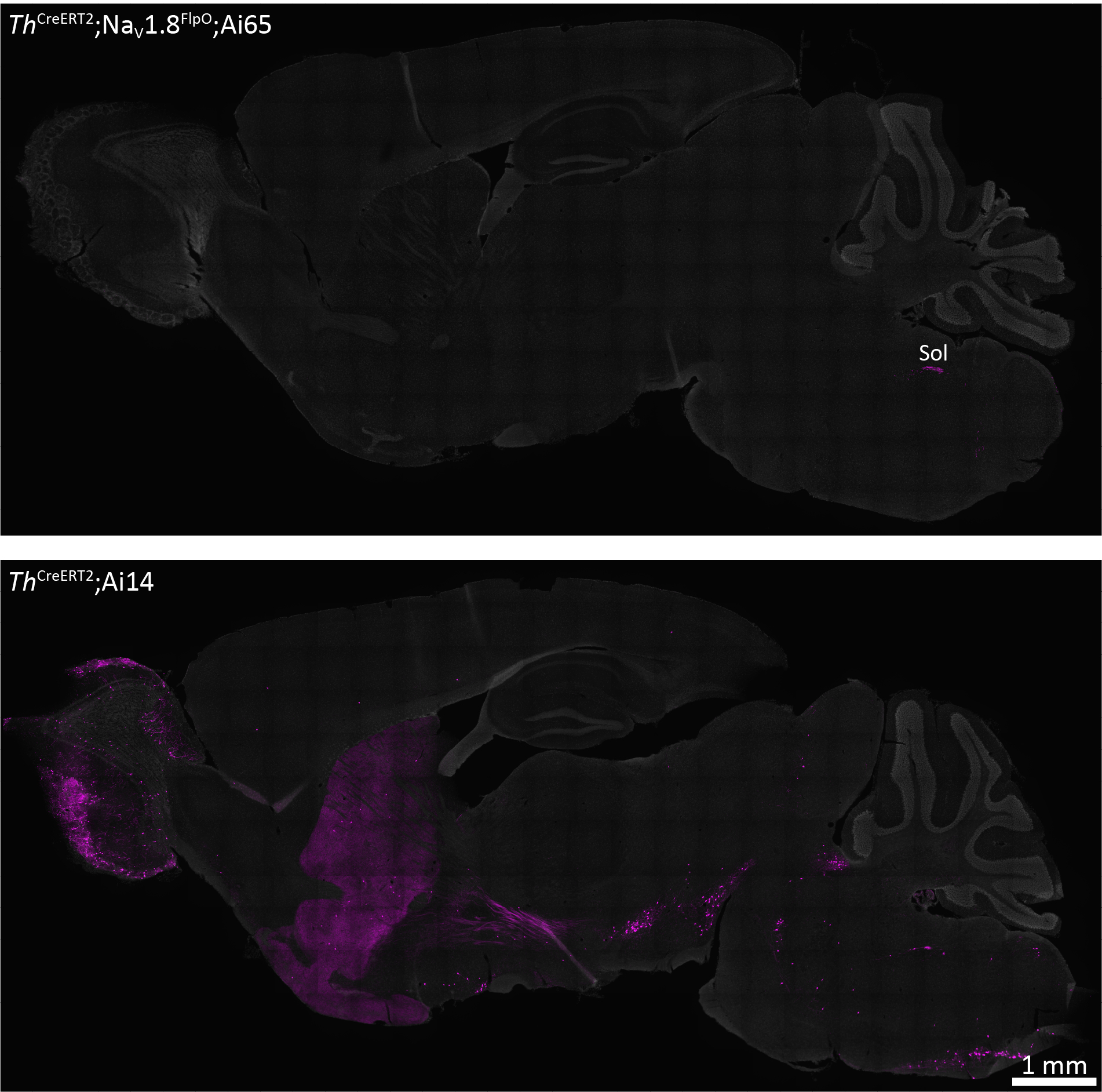

Supplement: SUPPLEMENTARY FIGURE S3 — tdTomato+ fibers and cells in the brains of ThCreERT2; NaV1.8FlpO; Ai65 and ThCreERT2; Ai14 mice. Shown are parasagittal brain sections displaying complete lack of tdTomato fluorescence in the brain of ThCreERT2;NaV1.8FlpO;Ai65 mice except for the nucleus of the solitary tracy (Sol) and very sparse fibers in trigeminal areas (top panel), and the expected wide distribution of catecholaminergic structures in ThCreERT2; Ai14 mice (bottom panel). Scale bar is 1 mm, valid for both panels. Images are widefield tilescan micrographs obtained using a 20x/0.5 objective. [file Image_3.jpg]
